# YTHDF2 Inhibits Gastric Cancer Cell Growth by Regulating FOXC2 Signaling Pathway

**DOI:** 10.3389/fgene.2020.592042

**Published:** 2021-01-11

**Authors:** Xudong Shen, Kui Zhao, Liming Xu, Guilian Cheng, Jianhong Zhu, Lei Gan, Yongyou Wu, Zhixiang Zhuang

**Affiliations:** ^1^Department of Oncology, The Second Affiliated Hospital of Soochow University, Suzhou, China; ^2^Department of General Surgery, The Second Affiliated Hospital of Soochow University, Suzhou, China; ^3^Department of Gastroenterology, The Second Affiliated Hospital of Soochow University, Suzhou, China

**Keywords:** gastric cancer, cell growth, YTHDF2, FOXC2, M6A

## Abstract

**Background:**

Gastric cancer (GC) is one of the most common malignancies in the world, and the fourth most frequent malignancy worldwide. YTHDF2 (YTH domain family 2, YTHDF2) binds to mRNA containing m6A, thereby regulating the localization and stability of the bound mRNA. YTHDF2 was shown to be associated with some cancer patient prognosis. However, the effect of YTHDF2 on gastric cancer and the molecular mechanism of this effect have not been documented.

**Methods:**

To conduct this research, YTHDF2 expression levels in public databases and gastric cancer patient samples were analyzed. The effects of YTHDF2 on the growth of gastric cancer cells were detected *in vivo* and *in vitro.* RNA-seq was used to analyze the signal pathways regulated by YTHDF2, and experiments were carried out for verification.

**Results:**

In our study, we found that YTHDF2 has lower expression in GC tissues and GC cells, and inhibits the growth of GC cells. In addition, the analysis of clinical data found that the expression level of YTHDF2 is closely related to the stage of GC and the survival of patients with GC. RNA sequencing results showed that overexpression of YTHDF2 significantly reduced protein expression in the FOXC2 (Forkhead box protein C2, FOXC2) signaling pathway. Finally, we found that knockout of FOXC2 reversed the inhibitory effect of YTHDF2 on GC cells.

**Conclusion:**

In summary, YTHDF2 inhibits the growth of GC cells by negatively regulating FOXC2 and may serve as a prognostic marker in GC.

## Introduction

Gastric cancer (GC) is a tumor with high morbidity and mortality (Bray et al., 2018). Currently, GC is the second most commonly diagnosed cancer in China (Chen et al., 2016). There are multiple chemotherapy and targeted drug treatment options for GC patients, but their metastasis, recurrence, and 5-year survival rates are still disappointing (Lambert et al., 2012). Therefore, the pathological and molecular mechanisms related to the progression of GC still need in-depth research.

N6-methyladenosine (m6A) is one of the most common internal modifications in the mRNAs of all higher eukaryotes (Wang et al., 2014). m6A regulates eukaryotic mRNA to affect its splicing, localization, translation, output, and stability, affecting a variety of basic biological functions and diseases (Jia et al., 2011). Current research has found that m6A methylation levels are closely related to a variety of tumors (Deng et al., 2018). YTHDF2, as part of the m6A-specific reader YTH domain family, is closely related to tumors. In pancreatic cancer, YTHDF2 regulates the EMT (epithelial-mesenchymal transition, EMT) process of pancreatic cancer cells through the YAP signaling pathway (Chen et al., 2017). YTHDF2 promotes the growth of lung cancer cells by promoting the translation of 6-phosphate gluconate dehydrogenase mRNA (Sheng et al., 2019). However, YTHDF2 reduces inflammation and vascular abnormalities in hepatocellular carcinoma by degrading mRNAs of m6A-modified interleukin 11 and serine protease inhibitor E family 2 (Hou et al., 2019). In addition, it has been reported that YTHDF2 significantly disrupts the mRNA stability of ERGF and inhibits the proliferation and growth of hepatocellular carcinoma cells (Zhong et al., 2019). In summary, YTHDF2 regulates the progression of different tumors by affecting the mRAN stability of tumor suppressor genes or oncogenes. However, the specific functions and underlying mechanisms of YTHDF2 in GC are not fully understood.

FOXC2 belongs to the Forkhead transcription factor family and was originally discovered during embryonic development (Iida et al., 1997). Abnormal FOXC2 expression was found in tumorigenesis and malignant tumor development. High expression of FOXC2 has been found in many tumors, such as nasopharyngeal cancer (Nishida et al., 2011) and triple negative breast cancers (Hollier et al., 2013). In addition, FOXC2 expression is closely related to poor prognosis of breast cancer (Bollong et al., 2017). In breast cancer, FOXC2 is involved in regulating the EMT process and breast cancer stem cells (Hollier et al., 2013). In colorectal cancer, FOXC2 also induces EMT through MAPK/ERK signaling, thereby enhancing oxaliplatin resistance (Chen et al., 2020). Analysis of GC clinical patients found that abnormal overexpression of FOXC2 was significantly associated with differentiation, depth of invasion, lymph node metastasis, tumor staging, and patient prognosis (Zhu et al., 2013). Although FOXC2 is closely related to GC metastasis and patient survival, its function in GC and its regulated mechanism are still unclear.

In this study, we examined GC tissue chips and clinical samples and found that YTHDF2 expression was significantly lower than that of normal gastric tissue. Further analysis revealed that the low expression of YTHDF2 was closely related to the clinical stage of GC and patient survival. GC cells over-expressed or knocked out YTHDF2 can, respectively, significantly inhibit or promote cell proliferation. Finally, we found that overexpression of YTHDF2 significantly inhibited the FOXC2 protein table by using RNA-Seq experiments, and inhibited GC cell growth by negatively regulating FOXC2 expression levels. Taken together, our data reveal that YTHDF2 inhibits the progression of GC by regulating FOXC2, providing a potential therapeutic target for clinical treatment of GC.

## Materials and Methods

### Cell Lines

Gastric cancer cell lines (SGC-7901, MKN-45, AGS, MKN-28, and MGC-803) and human normal gastric mucosal epithelial cells (NGEC) were purchased from ATCC (American Type Culture Collection, ATCC). NGEC, AGS cells were cultured in 90% DMEM (Biological Industries, Cat. No. 06-1055-57-1A), 10% FBS (Biological Industries, Cat. No. 04-007-1A), and 1% penicillin/streptomycin. SGC-7901, MKN-45, MKN-28, and MGC-803 cells were cultured in 90% RPMI (Biological Industries, Cat. No. 01-101-1A), 10% FBS, and 1% penicillin/streptomycin. Although MGC-803 cell reports show cross-contamination with HeLa, there are still many research groups using MGC-803 as a model for studying gastric cancer cells (Zhai et al., 2019; Li et al., 2020; Peng et al., 2020).

### Antibodies and Plasmids

YTHDF2 antibody (Abcam, ab220163), FOXC2 antibody (ab24340), GSK-3β antibody (Abcam, ab93926), and Snail antibody (Abcam, ab229701) were purchased from Abcam. Over-expressed YTHDF2 lentiviral plasmid was purchased from GeneCopoeia. The shYTHDF2 plasmid was commissioned by Shanghai Genechem Co., Ltd. The sequence of shYTHDF2 is as follows: shYTHDF2 # 1 CCGGGCTACTCTGAGGACGATATTCCTCGAGGAATATCG TCCTCAGAGTAGCTTTTTG; shYTHDF2 # 2 CCGGTACTG ATTAAGTCAGGATTAACTCGAGTTAATCCTGACTTAATCA GTATTTTTG. The lentiviral system was used to package YTHDF2 or shYTHDF2 lentiviral particles, and then virally infect the gastric cancer cell lines. The finally established overexpression or knockout YTHDF2 cell line had undergone long-term puromycin screening.

### qRT-PCR

Gastric cancer cells that overexpress or knock out YTHDF2 were extracted with total RNA by Trizol lysis. CDNA was obtained using a reverse transcription kit. QRT-PCR was performed using the following primers: 5′- ATAGTTTGCCTCCAGCCACC -3′ (YTHDF2 Forward) and 5′- GGACCGAAGCTTCTCCAACA -3′ (YTHDF2 Reverse). The relative expression level of YTHDF2 is the minus 2 power of the difference between the cycle number of YTHDF2 and the cycle number of GAPDH.

### Western Blot

Gastric cancer cells overexpressing or knocking out YTHDF2 and each gastric cancer cell line were subjected to RIPA to extract total protein. The protein was electrophoresed on SDA-PAGE gel, transferred to a mold, blocked, and incubated with antibodies. Protein bands were acquired using BioImaging Systems and protein gray values were counted using Image J.

### MTT

Gastric cancer cell lines with the appropriate density of overexpression or knockout of YTHDF2 were seeded in 96-well plates. The cells were cultured for 24, 48, and 72 h, and then added with MTT (0.5 mg/ml) and incubated for 4 h. DMSO was then added and the OD value was measured using a spectrophotometer.

### Clonogenicity

Over-expressed or knocked out YTHDF2 gastric cancer cell lines of appropriate density were mixed with agar and seeded in 6-well plates. Fresh media was added three times a week and photographed and counted after 14 days.

### Immunohistochemistry

Gastric cancer tissue and heterogeneous tumor tissue sections were sequentially dewaxed and repaired with citric acid antigen. The tissue sections were then treated with 3% hydrogen peroxide, followed by serum blocking and antibody incubation (YTHDF2, ab246514, 1/2,000 dilution; Ki67, ab92742, 1/1,000 dilution). Image-Pro software was used to analyze YTHDF2 tumor tissue expression.

### *In vivo* Tumorigenesis

A large number of over-expressed or knocked out YTHDF2 gastric cancer cell lines were seeded in nude mice to construct heterogeneous tumor models. Heterogeneous tumors in nude mice were measured once a week. After 42 days of breeding the nude mice, the heterogeneous tumor mass was removed from the mice, and the tumor mass size was measured and weighed.

### RNA-Seq Analysis

MGC-803 cells overexpressing YTHDF2 were collected and total RNA was extracted using Trizonl. RNA sequence analysis was performed on total RNA, and a library was prepared using IlluminaTruSeq1 RNA sample preparation v2. The samples were sequenced using Illumina HiSeq 2500 single-read mode and the sequencing depth was set to 50 times. HTSeq (version 0.6.1) was used to quantify gene-level expression, and differential expression was calculated using DESeq2 software (version 1.2.10).

### Bioinformatic Analysis

GSE103236, GSE118897, and GSE118916 public data were obtained from the oncomine^1^ database. GISTIC database was used to analyze the level of YTHDF2 gene copy number in gastric cancer patients.

### Gastric Cancer Patient Samples

**FIGURE 1:**
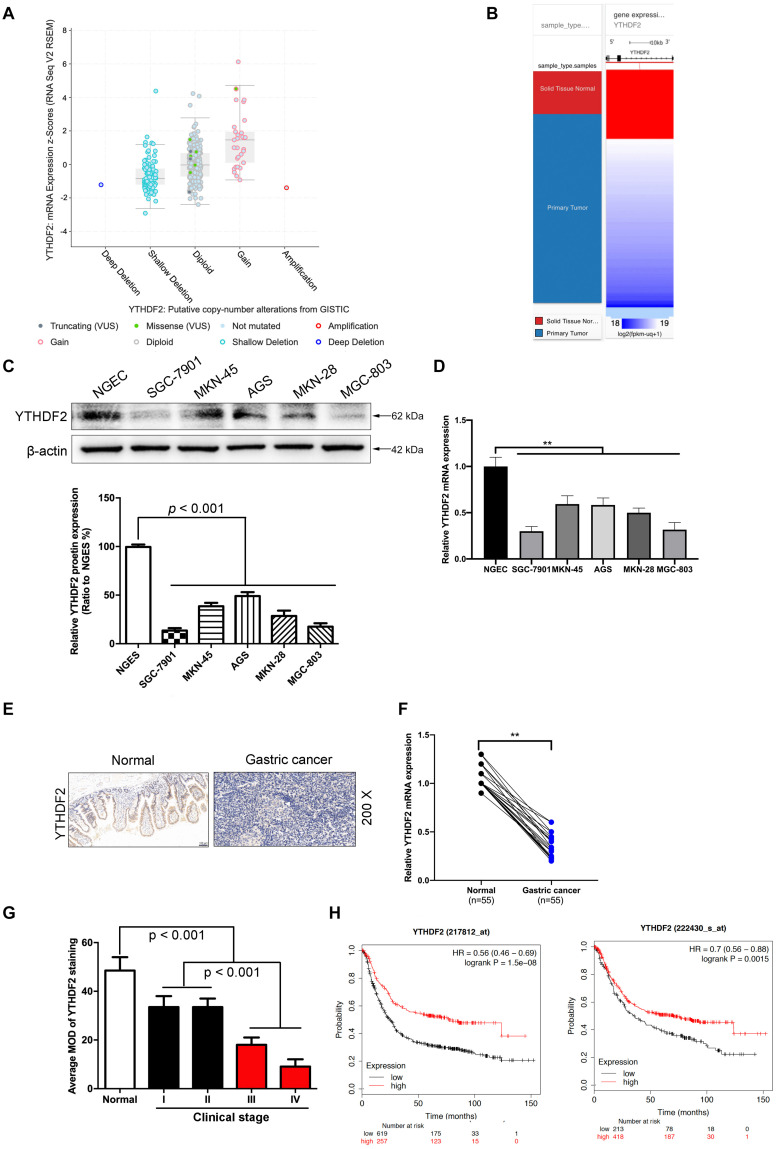
The low expression level of YTHDF2 in gastric cancer is closely related to the tumor. **(A)** GISTIC database analyzed the copy number of YTHDF2 in gastric cancer tissues. **(B)** Analysis of the expression level of YTHDF2 in gastric primary cancer. **(C,D)** Western blot and qRT-PCR analysis of the expression of YTHDF2 protein in gastric cancer cells and normal gastric epithelial cells. **(E)** Immunohistochemical staining of representative gastric cancer tissues. **(F)** YTHDF2 mRNA expression levels in gastric cancer and Normal tissue; **(G)** YTHDF2 expression in gastric cancer of different clinical stages. **(H)** Analysis of the relationship between YTHDF2 and survival of gastric cancer patients. Data are the means ± *SD* of three **(F)** independent experiments. The scale bars indicate 100 μm in **(E)**. **p < 0.01. Data are the means ± *SD* of three independent experiments.

We commissioned the Department of Pathology, the Second Affiliated Hospital of Soochow University to collect clinical samples of gastric cancer. Our use of clinical samples from gastric cancer patients meets the approval of the Ethics Committee of the Second Affiliated Hospital of Soochow University (Approval number: ECSSCU2018-2-002). The YTHDF2 expression level in clinical samples from the gastric cancer database^2^ was divided into four equal parts (bottom quartile, mid-low quartile, mid-high quartile, and top quartile), and survival analysis (Figure 1H) was performed on the bottom quartile and top quartile.

### Statistical Analysis

Data were analyzed using the Student’s *t*-test by SPSS. Date are presented as mean ± *SD*, and *p* < 0.05 were considered statistically significant.

## Results

### The Low Expression Level of YTHDF2 in Gastric Cancer Is Closely Related to the Tumor Stage

In order to explore how YTHDF2 plays a role in GC, we first analyzed the expression of YTHDF2 in GC patients from public data. In the GC gene chip (GSE103236, GSE118897, and GSE118916) in public data, we found that the YTHDF2 gene has significantly lower expression in the GC group compared to normal gastric tissue (Supplementary Figures 1A–C). Simultaneously, we also used GISTIC data to analyze the copy number of YTDF2 in GC patients. The results showed that the copy number of YTHDF2 was generally lower in patients with GC (Figures 1A,B). In addition, we also detected the protein expression level of YTHDF2 in GC cell lines and normal gastric epithelial cells by Western blot and qRT-PCR. The results showed that, compared with normal gastric epithelial cells, YTHDF2 was significantly lower in GC cell lines (Figures 1C,D). Further immunohistochemical was performed to detect the expression level of YTHDF2 in GC tissue. The results also showed that YTHDF2 was at a low expression level in GC tissue (Figures 1E,F). Finally, we also analyzed the role of YTHDF2 in the clinical stage and prognosis of GC. The clinical staging results of GC patients showed that the expression level of YTHDF2 was significantly correlated with clinical analysis, and the expression level of YTHDF2 was lower in the tissues with a higher malignant degree of GC (Figure 1G and Supplementary Figure 1D). The expression level of YTHDF2 was also related to the survival time of patients. The survival time of patients with low YTHDF2 expression is significantly lower than that of patients with high YTHDF2 expression (Figure 1H). These results indicate that the low expression of YTHDF2 plays an important role in the stage and survival of GC.

### Overexpression of YTHDF2 Inhibits Gastric Cancer Cell Growth and Malignancy

**FIGURE 2:**
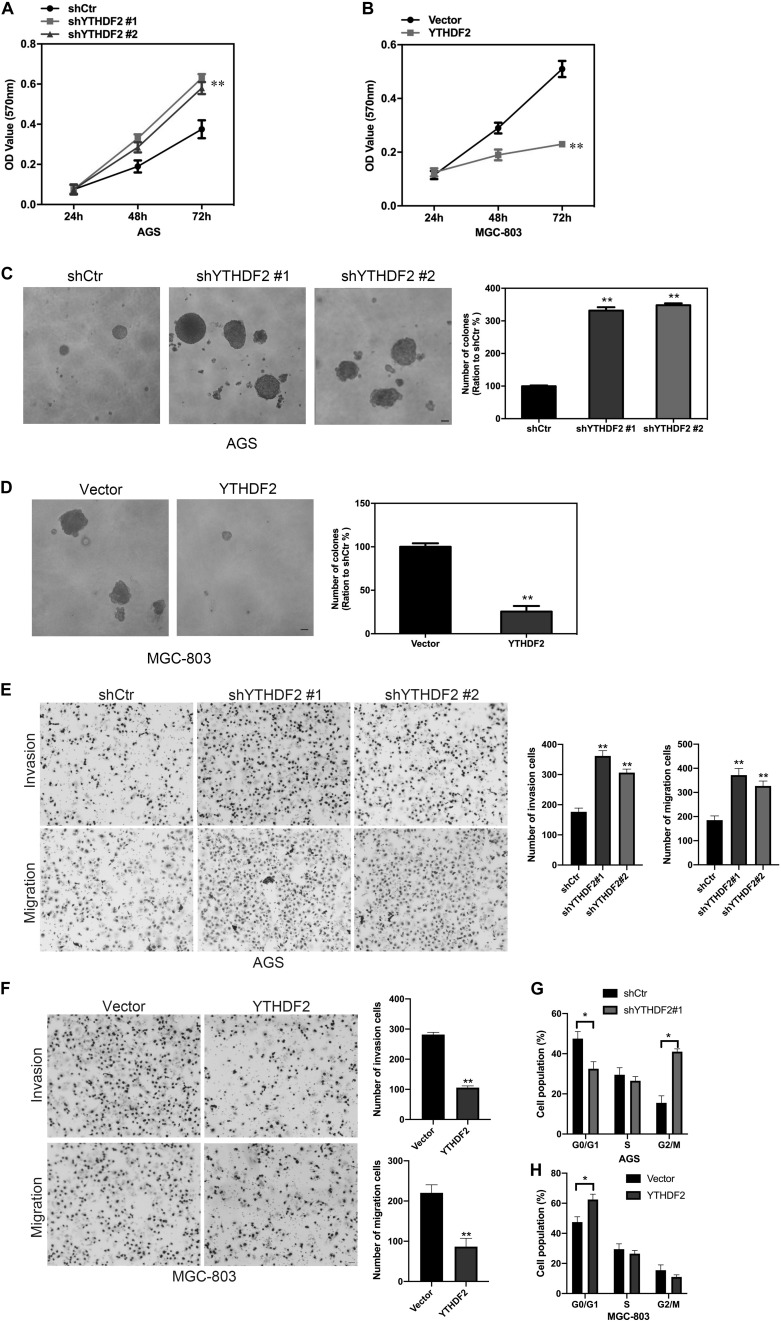
Effect of YTHDF2 on the proliferative ability of gastric cancer cells *in vitro*. **(A,B)** MTT detected the effect of over-expression or knockout of YTHDF2 on the cell viability of gastric cancer cells. **(C,D)** Clone formation was used to detect the effects of over-expression or knockout of YTHDF2 on the viability of gastric cancer cells. **(E,F)** The effect of overexpression or knockout of YTHDF2 on cell migration and invasion. **(G,H)** The effect of overexpression or knockout of YTHDF2 on cell cycle. *p < 0.05, **p < 0.01. Data are the means ± *SD* of three independent experiments. The scale bars indicate 20 μm in **(C–E)**.

We have found that YTHDF2 is under-expressed in GC and has implications for GC stage and prognosis. Next, we will explore the function of YTHDF2 in GC. To further determine the effect of YTHDF2 on GC cells, we constructed YTHDF2 knockout cell lines in AGS and MKN-45 cells (Supplementary Figures 2A,B,E,F), and over-expressed YTHDF2 cell lines in MGC-803 and SGC-7901 cells (Supplementary Figures 2C,D,G,H). Using the above cell lines, the effect of YTHDF2 on the cell viability of GC cells was examined by MTT. MTT results showed that knockout of YTHDF2 clearly increased the cellular activity of AGS and MKN-45 cells (Figure 2A and Supplementary Figure 3A), while overexpression of YTHDF2 significantly inhibited the cellular activity of MGC-803 and SGC-7901 cells (Figure 2B and Supplementary Figure 3B). Furthermore, we used soft agar to examine the proliferation ability of YTHDF2 on GC cells. The results showed that knockout YTHDF2 significantly increased the proliferation ability of AGS and MKN-45 cells (Figure 2C and Supplementary Figure 3C), while overexpression of YTHDF2 significantly inhibited the proliferation ability of MGC-803 and SGC-7901 cells (Figure 2D and Supplementary Figure 3D). In addition, we also tested the effect of YTHDF2 on the migration and invasion of gastric cancer cells. The results showed that knocking out YTHDF2 details increased the invasion and migration of GC cells (Figure 2E and Supplementary Figure 3E), while overexpression of YTHDF2 significantly inhibited the invasion and migration of GC cells (Figure 2F and Supplementary Figure 3F). Finally, we also found that overexpression of YTHDF2 significantly increased cell cycle blockage in G1 phase (Figure 2G), while knocking out YTHDF2 significantly increased the ratio of cells in G2/M phase (Figure 2H).

**FIGURE 3:**
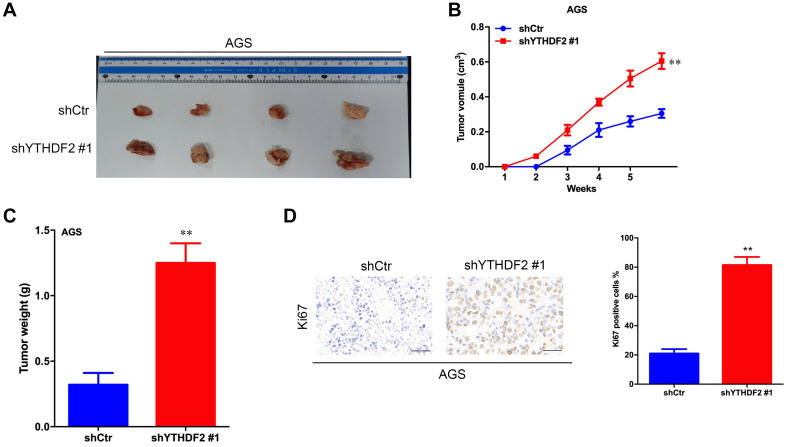
Effect of knockout of YTHDF2 on gastric cancer cell proliferation ability *in vivo*. **(A)** The size of heterogeneous tumors of AGS cells. **(B)** The growth rate of heterogeneous tumors of AGS cells. **(C)** The weight of heterogeneous tumors of AGS cells. **(D)** The expression of YTHDF2 in AGS heterogeneous tumors by histochemical staining. **p < 0.01. Data are the means ± *SD*. The scale bars indicate 50 μm in **(D)**.

**FIGURE 4:**
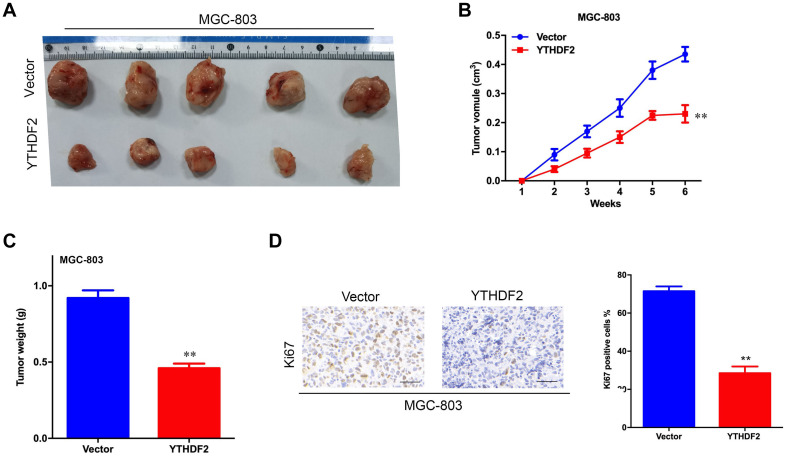
Effect of overexpression of YTHDF2 on gastric cancer cell proliferation ability *in vivo*. **(A)** The size of heterogeneous tumors of MGC-803 cells. **(B)** The growth rate of heterogeneous tumors of MGC-803 cells; **(C)** The weight of heterogeneous tumors of MGC-803 cells. **(D)** The expression of YTHDF2 in MGC-803 heterogeneous tumors by histochemical staining. **p < 0.01. Data are the means ± *SD*. The scale bars indicate 50 μm in **(D)**.

The results of *in vitro* cell-level experiments show that YTHDF2 has the ability to inhibit the proliferation of GC cells, so does it have the same effect *in vivo*? A nude mouse heterogeneous tumor model was used to detect the effect of YTHDF2 on subcutaneous tumor formation of GC cells. The experimental results showed that the growth volume and weight of AGS cells with YTHDF2 knockout were significantly higher than those of the control group (Figures 3A–C). In addition, histochemical staining of the tumor confirmed the low expression of YTHDF2 in the tumor tissue (Figure 3D). The growth rate and weight of MGC-803 cells overexpressing YTHDF2 were significantly lower than those of the control group (Figures 4A–C). Immunohistochemical staining was performed on the tumor body of MGC-803 to verify the expression of YTHDF2 (Figure 4D).

These results show that YTHDF2 has the ability to inhibit the proliferation of GC cells, which in turn affects the clinical stage of GC and the survival of patients.

### YTHDF2 Regulates Gastric Cancer Cell Proliferation Through FOXC2 Signaling Pathway

**FIGURE 5:**
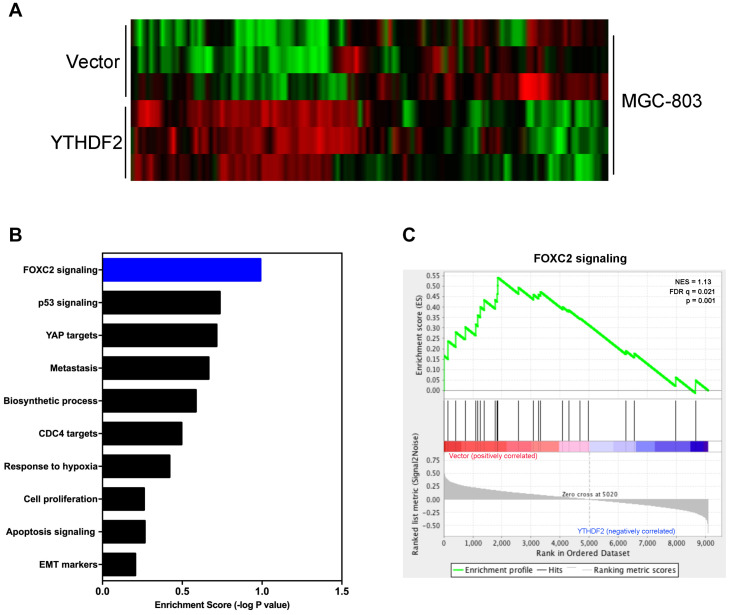
YTHDF2 regulates FOXC2 signaling pathway. **(A)** Heat map analysis of YTHDF2 regulated genes; **(B)** KEGG analysis of YTHDF2 regulated genes’ signal pathways. **(C)** Analysis of YTHDF2 regulated FOXC2 signaling pathways.

Next, we explored the relevant mechanism of YTHDF2’s inhibitory effect on GC cell proliferation. We used MGC-803 cells overexpressing YTHDF2 for RNA sequencing to find possible regulatory mechanisms. We used MGC-803 cells overexpressing YTHDF2 for RNA sequencing to find possible regulatory mechanisms. Sequencing results showed genes with differential changes (Figure 5A). KEGG signal pathway analysis was performed on the different genes, and multiple tumor-related signal pathways were found (Figure 5B). Among them, the most obvious difference was the FOXC2 signaling pathway, which had a high functional enrichment (Figure 5C).

**FIGURE 6:**
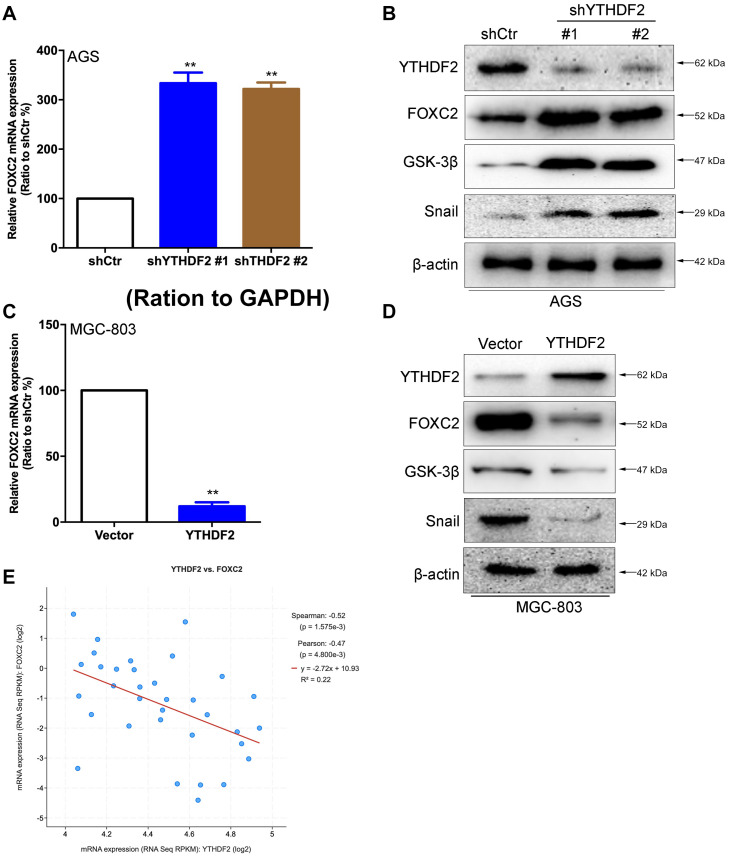
YTHDF2 negatively regulates FOXC2 expression level. **(A)** qRT-PCR detection of FOXC2 expression in AGS cell lines with knockout of YTHDF2. **(B)** Western blot detected FOXC2, GSK-3β, and Snail protein expression levels in AGS cell lines that knocked out YTHDF2. **(C)** qRT-PCR detection of FOXC2 expression in MGC-803 cell line overexpressing YTHDF2. **(D)** Western blot was used to detect the expression of FOXC2, GSK-3β, and Snail protein in MGC-803 cell line overexpressing YTHDF2. **(E)** Analysis of the regulatory relationship between YTHDF2 and FOXC2 in public data on gastric cancer. **p < 0.01. Data are the means ± *SD* of three **(A,C)** independent experiments.

In order to verify the relationship between YTHDF2 and the FOXC2 signaling pathway, we first verified that YTHDF2 negatively regulates FOXC2. Using the AGS cell line that knocked out YTHDF2, qRT-PCR and Western blot results showed that FOXC2, GSK-3β, and Snail expression levels were significantly increased compared to the control group (Figures 6A,B). Simultaneously using MGC-803 cell line over-expressing YTHDF2, qRT-PCR and Western blot results showed that the expression level of FOXC2, GSK-3β, and Snail were significantly reduced compared to the control group (Figures 6C,D). In addition, we also analyzed the expression level between YTHDF2 and FOXC2 in the public database. The results showed that the expression level of YTHDF2 was negatively correlated with the expression level of FOXC2 (Figure 6E).

### YTHDF2 Regulates the Stability of FOXC2 mRNA and Inhibits the Proliferation and Migration of Gastric Cancer Cells

**FIGURE 7:**
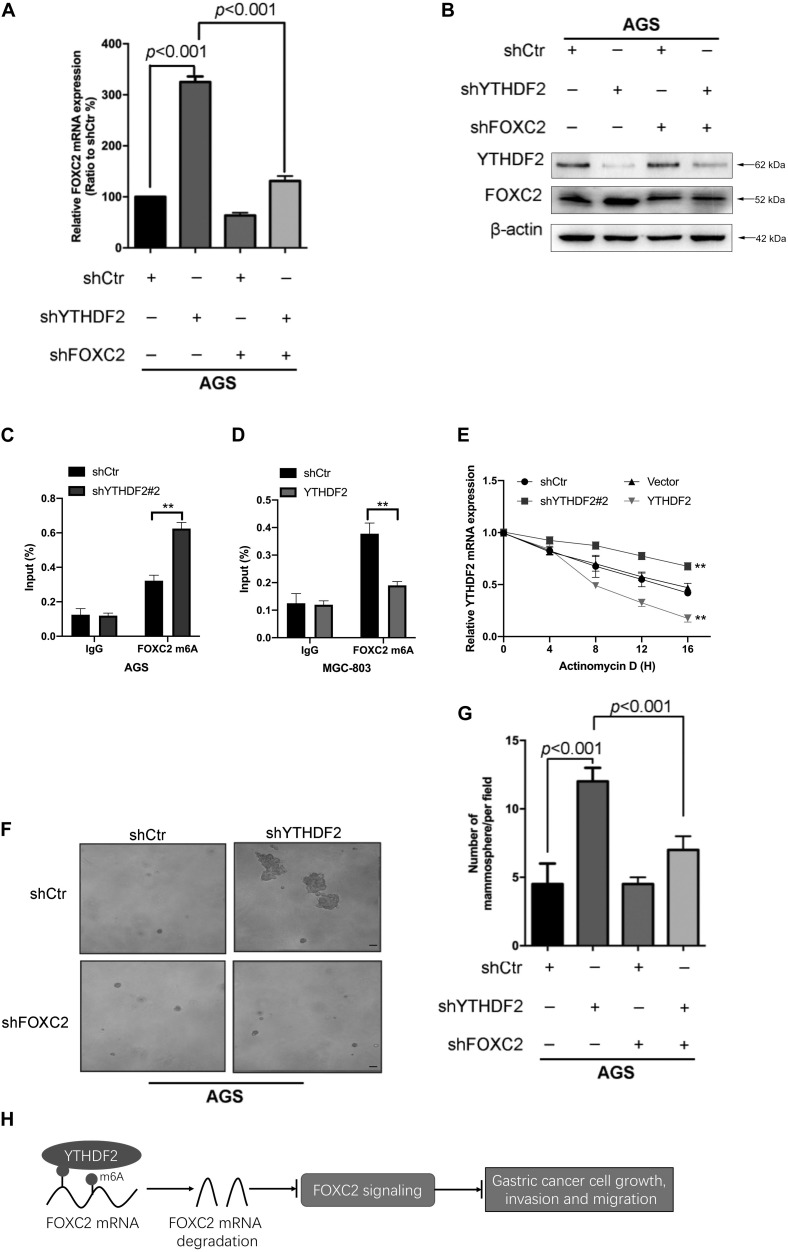
YTHDF2 regulates gastric cancer cell proliferation by regulating the stability of FOXC2 mRNA. **(A,B)** qRT-PCR and Western blot to verify the knockout efficiency of YTHDF2 or FOXC2; **(C,D)** RIP-qPCR analysis of m6A modification of FOXC2 mRNA in cells overexpressing or knocking out YTHDF2. **(E)** Actinomycin D treated with overexpression or knockout YTHDF2 cells to detect FOXC2 mRNA stability. **(F,G)** Clone formation detected the effects of knockout of YTHDF2 and FOXC2 on the proliferation ability of gastric cancer cells. **(H)** YTHDF2 regulates gastric cancer cell proliferation, invasion, and migration by regulating the stability of FOXC2 mRNA. **p < 0.01. Data are the means ± *SD* of three **(A,D)** independent experiments. The scale bars indicate 50 μm in **(F)**.

As reported in previous work, YTHDF2 induces target mRNA degradation by reading m6A modification sites (Wang et al., 2014). In order to test whether YTHDF2 regulates FOXC2 mRNA levels, we tested the FOXC2 expression level in AGS cells with YTHDF2 knockout. The results showed that when YTHDF2 was knocked out, the mRNA and protein levels of FOXC2 increased significantly (Figures 7A,B). Furthermore, we also tested the m6A level of YTHDF2 mRNA and found that in AGS cells with YTHDF2 knockout, the m6A level of FOXC2 was significantly increased (Figure 7C), while the opposite was true in MGC-803 cells with YTHDF2 knockout (Figure 7D). In actionomycin-treated cells, knocking out YTHDF2 significantly increased the stability of FOXC2 mRNA, while overexpression of YTHDF2 significantly reduced the stability of FOXC2 mRNA (Figure 7E). These results show that YTHDF2 regulates its mRNA stability by reading the m6A modification of FOXC2 mRNA. Further knocking out YTHDF2 and FOXC2 can reverse the inhibitory effect of knocking out YTHDF2 on ASG cell proliferation (Figures 7F,G). In addition, overexpression of YTHDF2 and FOXC2 in MGC-803 cells (Supplementary Figure 4A) and overexpression of FOXC2 can significantly inhibit YTHDF2’s inhibitory effects on cell proliferation, invasion, and migration (Supplementary Figures 4B–D).

These results show that YTHDF2 negatively regulates FOXC2 mRNA and thus inhibits the proliferation, invasion, and migration of GC cells (Figure 7H).

## Discussion

YTHDF2 is the most effective m6A reader (Wang et al., 2014). It weakens mRNA stability by recognizing m6A-modified mRNA and distributing it to processed bodies (Edupuganti et al., 2017). However, the function of YTHDF2 in GC is still controversial. In this study, we found that YTHDF2 had a low expression in GC and was closely related to the clinical stage of GC and patient survival. It was further found that over-expression of YTHDF2 significantly inhibited the proliferation, invasion, and migration of GC cells *in vitro* and *in vivo*. Finally, we found that YTHDF2 negatively regulated the expression level of the oncogene FOXC2 mRNA, thereby inhibiting the malignant behavior of GC cells.

YTHDF2, which belonged to a key reader, was the first method to degrade target mRNA or non-coding RNA by combining the m6A modification site with the G(m6A)C conserved core motif to achieve the target deletion phenotype (Wang et al., 2014). Specifically, the C-terminal domain of YTHDF2 selectively bound to the m6A modification site, and the N-terminal domain mediated the location of the YTHDF2-mRNA complex to the cellular RNA decay site by recruiting the CCR4-NOT complex (Du et al., 2016). Recent studies have found that YTHDF2 has the ability to degrade mRNAs of oncogenes and tumor suppressor genes in different tumors (Chen et al., 2018; Su et al., 2018; Zhong et al., 2019; Liu et al., 2020). It is precisely because YTHDF2 degrades oncogene or tumor suppressor gene mRNA that the function of YTHDF2 in tumorigenesis is still controversial. Studies have shown that YTHDF2 induced the targeted degradation of the tumor suppressor SOCS2, thereby promoting the tumor progression of hepatocellular carcinoma, and this regulation depended on the m6A modification induced by METL3 (Chen et al., 2018). However, other studies show that YTHDF2 suppresses cell proliferation and growth via destabilizing the EGFR mRNA in hepatocellular carcinoma (Zhong et al., 2019). In addition, reducing YTHDF2 reduced m6A-containing IL11 and SERPINE2 mRNA in hepatocellular carcinoma to aggravate inflammation and vascular abnormalities (Hou et al., 2019). In gastric cancer, studies have shown that the degradation of PTEN mRNA mediated by LINC00470-METTL3 depends on the m6A read protein YTHDF2-dependent pathway to promote the malignant behavior of gastric cancer cells (Yan et al., 2020). However, it is unclear whether YTHDF2 also regulates the mRNA stability of oncogenes in gastric cancer.

FOXC2 is a member of the FOXC subfamily and is involved in biological processes such as skeletal tissue development (You et al., 2014), adipocyte metabolism (Cederberg et al., 2001), and lymph angiogenesis (Wu and Liu, 2011). In addition, more evidence establishes the emerging role of FOXC2 in the development of cancer (Wang et al., 2018). They promote cancer by mediating cell proliferation, metastasis, and epithelial-mesenchymal transition (EMT) (Kume, 2008; Hollier et al., 2013). In our study, we found that YTHDF2 negatively regulates FOXC2 mRNA and protein expression, thereby inhibiting the malignant behavior of GC cells. We found that YTHDF2 recognizes the m6A of FOXC2 and degrades the mRNA level of FOXC2. It has been reported that LINC00470-METT3-mediated degradation of PTEN mRNA depends on YTHDF2. This difference may come from our studies that did not explore the upstream of the m6A modification of FOXC2 mRNA. The expression level of m6A “writer” in gastric cancer cells and the selectivity of mRNA modification may affect the ability of YTHDF to degrade the m6A mRNA of oncogenes or tumor suppressor genes. In future research, we will continue to explore which “writer” modifies the m6A modification of FOXC2 mRNA, which may help explain the difference between our findings and previous reports.

In summary, we have discovered a new potential mechanism for negatively regulating the expression level of FOXC2 in GC by YTHDF2, finding a reasonable mechanism for YTHDF2 to inhibit the proliferation, invasion, and migration of GC cells, and also finding new targets for the clinical treatment of GC.

## Data Availability Statement

The original contributions presented in the study are publicly available. This data can be found here: GEO accession number GSE162004.

## Ethics Statement

All animal experiments strictly abide by the relevant principles of the Animal Experimental Ethics Committee of the Second Affiliated Hospital of Soochow University.

## Author Contributions

XS, ZZ, and YW conceived and supervised the project, acquired funding, performed and designed the majority of experiments. XS, KZ, and LX wrote the manuscript. GC, JZ, and LG performed the experiments and supported the biochemical assays. All authors contributed to the article and approved the submitted version.

## Conflict of Interest

The authors declare that the research was conducted in the absence of any commercial or financial relationships that could be construed as a potential conflict of interest.

## Footnotes

1 https://www.oncomine.org/

2 http://kmplot.com/analysis/index.php?p=service&cancer=gastric
