# Supplementary material for: YTHDF2 Inhibits Gastric Cancer Cell Growth by Regulating FOXC2 Signaling Pathway
Source: Front Genet. 2021 Jan 11;11:592042. doi: 10.3389/fgene.2020.592042 (PMC7831514; doi:10.3389/fgene.2020.592042)

**Supplemental Figure**

**
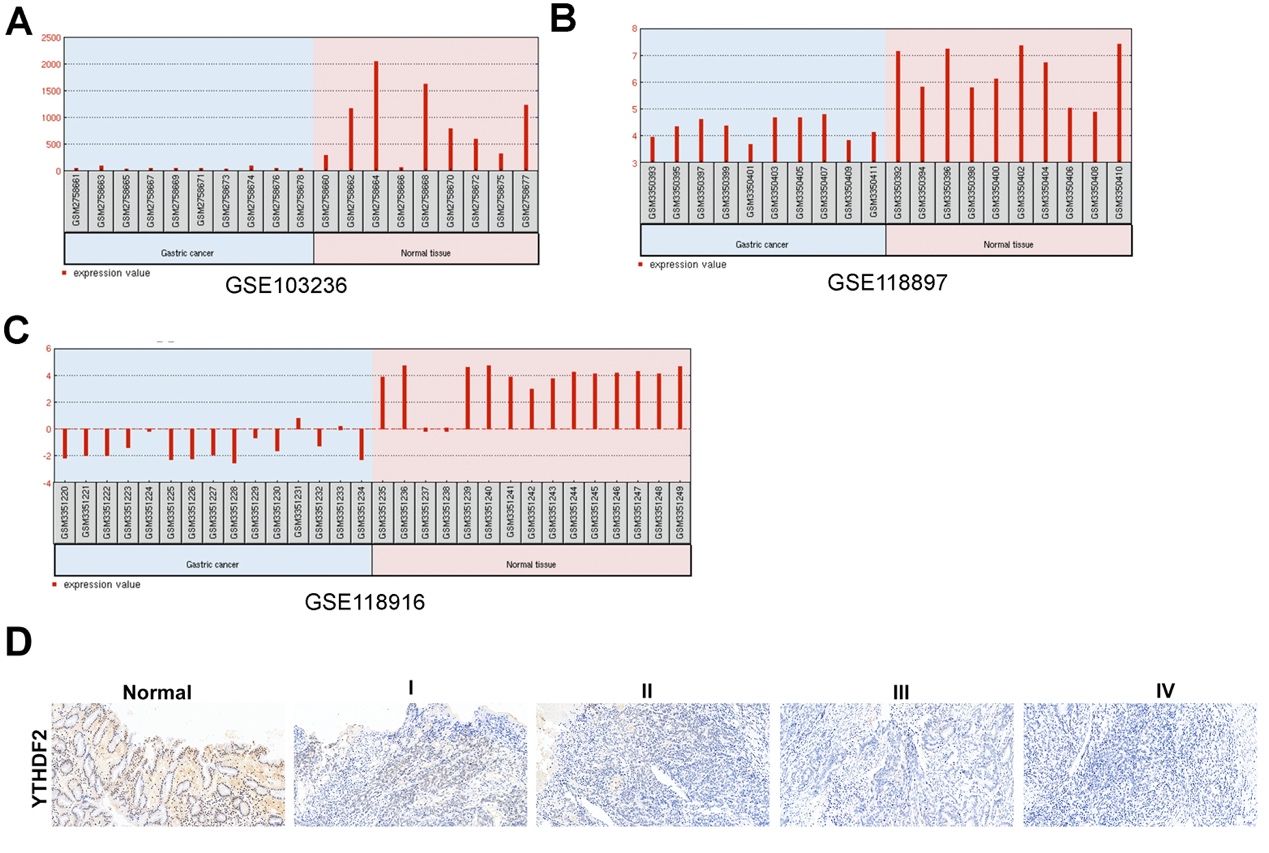
**

**Supplementary Fig. 1** (A, B, C) Gastric cancer public data analysis of YTHDF2 gene expression level; (D) Immunohistochemical staining of YTHDF2 in samples of different clinical stages of gastric cancer patients.

**
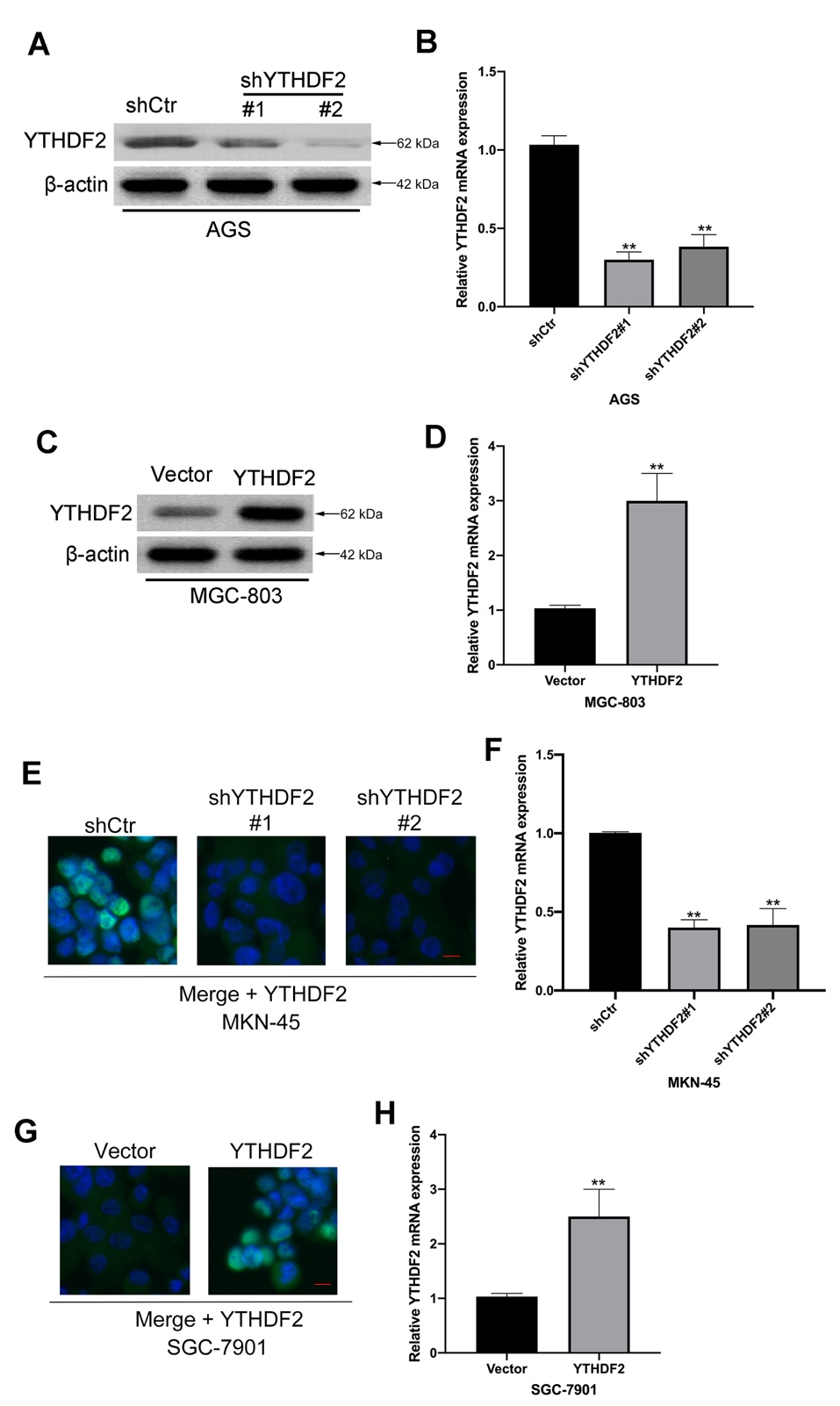
**

**Supplementary Fig. 2** Construct overexpression and knock out YTHDF2 in gastric cancer cells. (A, B) Western blot and qRT-PCR detection of YTHDF2 expression level in AGS cells knocked out YTHDF2; (C, D) Western blot and qRT-PCR detection of YTHDF2 expression level in MGC-803 cells overexpression YTHDF2; (E) Immunofluorescence detection of YTHDF2 expression level in MKN-45 cells with YTHDF2 knockout; (F) qRT-PCR detection of YTHDF2 expression level in MKN-45 cells with YTHDF2 knockout; (G) Immunofluorescence detection of YTHDF2 expression level in SGC-7901 cells with YTHDF2 overexpression; (H) qRT-PCR detection of YTHDF2 expression level in SGC-7901 cells with YTHDF2 overexpression; ***p*< 0.01. Data are the means ± SD of three (A, B) independent experiments.


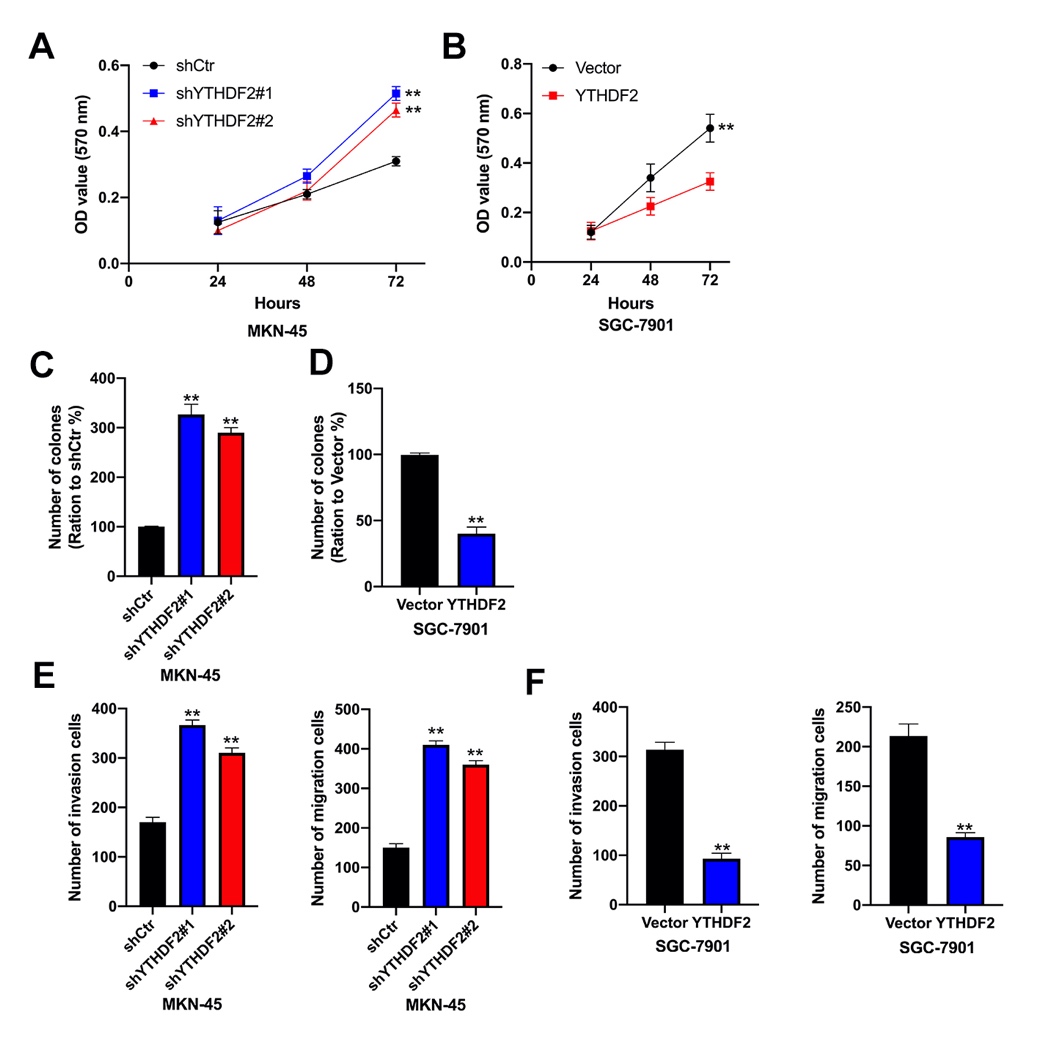


**Supplementary Fig. 3** Effect of YTHDF2 on the proliferative ability of gastric cancer cells *in vitro*. (A, B) MTT detected the effect of over-expression or knockout of YTHDF2 on the cell viability of gastric cancer cells; (C, D) Clone formation was used to detect the effects of over-expression or knockout of YTHDF2 on the viability of gastric cancer cells; (E, F) The effect of overexpression or knockout of YTHDF2 on cell migration and invasion. ***p*< 0.01. Data are the means ± SD of three independent experiments.


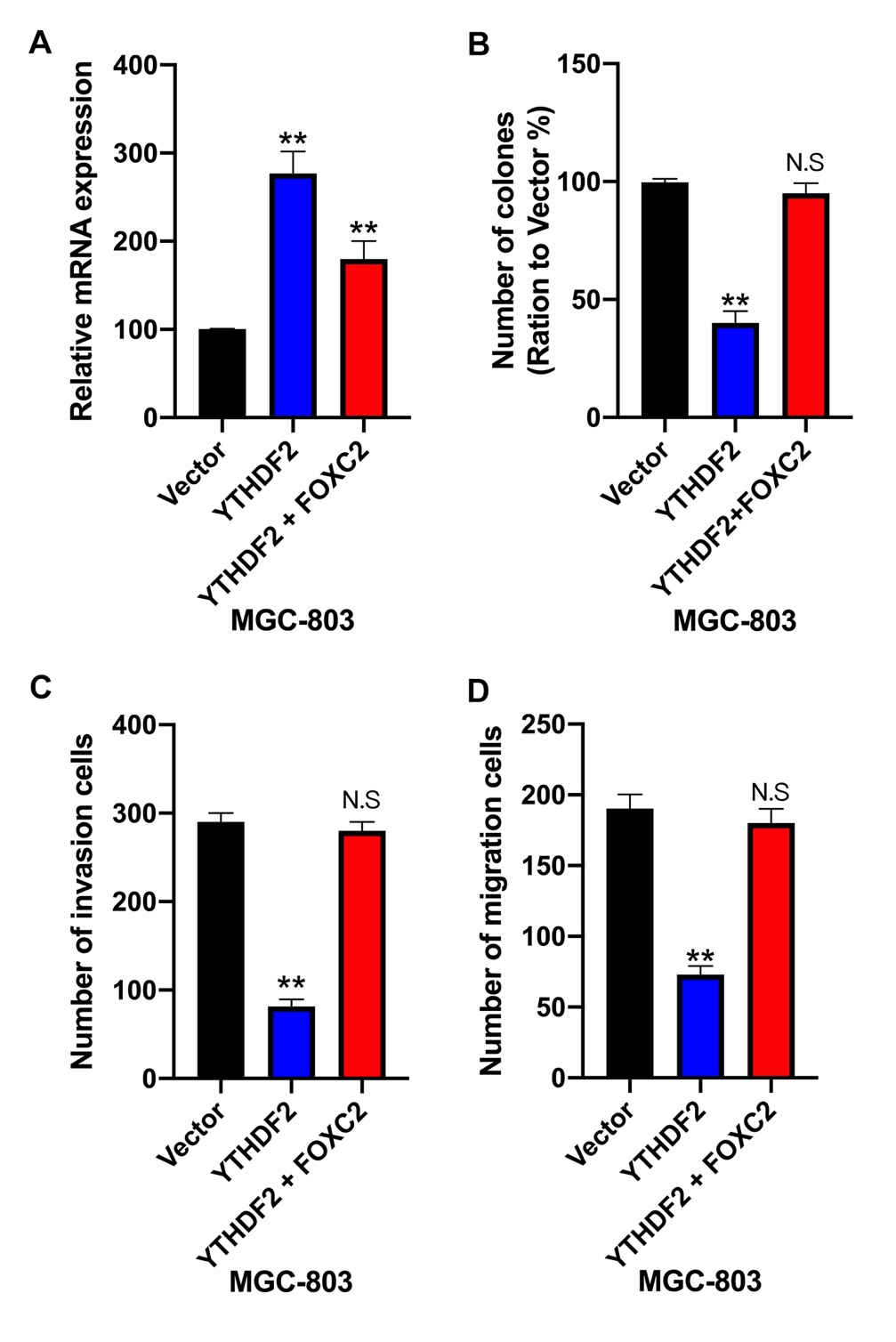


**Supplementary Fig. 4** Overexpression of FOXC2 reversed the inhibitory effect of YTHDF2 on gastric cancer cells. (A) MGC-803 cell line overexpressed YTHDF2 and FOXC2; (B) MGC-803 cells overexpressing YTHDF2 and FOXC2 were tested for cell clone formation ability; (C) MGC-803 cells overexpressing YTHDF2 and FOXC2 were tested Cell migration and invasion ability. ***p*< 0.01, N.S = No difference. Data are the means ± SD of three independent experiments.

**Original Western blot image**


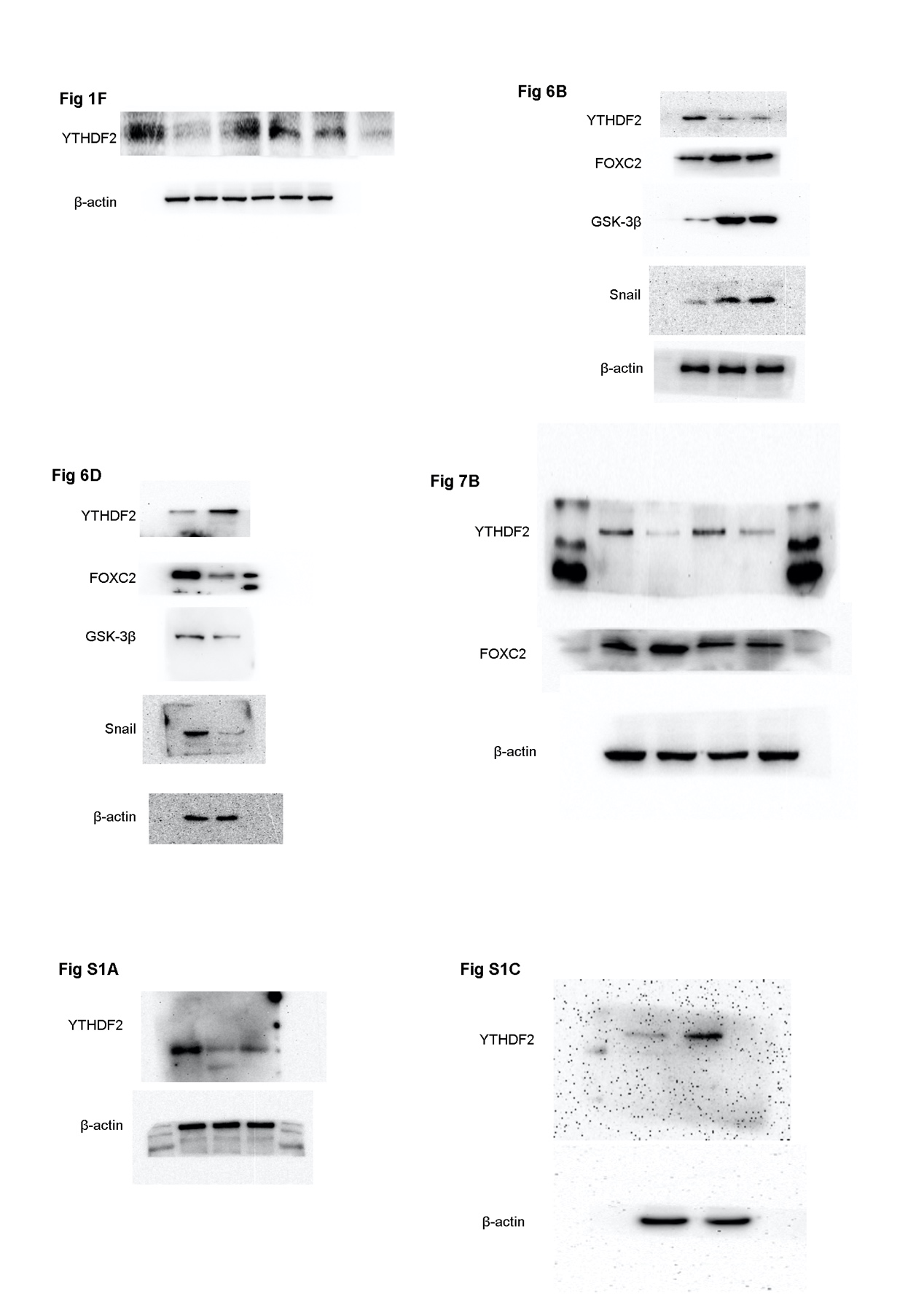

Supplement: Supplementary file 1 [file Data_Sheet_1.docx]
